# Circular RNA TLK1 Promotes Sepsis-Associated Acute Kidney Injury by Regulating Inflammation and Oxidative Stress Through miR-106a-5p/HMGB1 Axis

**DOI:** 10.3389/fmolb.2021.660269

**Published:** 2021-06-25

**Authors:** Hai-Ping Xu, Xiao-Ying Ma, Chen Yang

**Affiliations:** Department of Nephrology II, Cangzhou Central Hospital, Cangzhou, China

**Keywords:** sepsis, acute kidney injury, CircTLK1, MiR-106a-5p, HMGB1

## Abstract

Sepsis is an inflammatory disorder and leads to severe acute kidney injury (AKI). Circular RNAs (circRNAs) have been identified as a critical type of regulatory noncoding RNAs (ncRNAs) that present the important functions in various diseases. In this study, we identified a novel circRNA circTLK1 in the regulation of sepsis-induced AKI. We observed that circTLK1 expression was elevated in the cecal ligation and puncture (CLP) rat model compared with that in the control rats. The urine levels of neutrophil gelatinase–associated lipocalin (NGAL) and kidney injury molecule-1 (Kim-1) and the serum levels of creatinine (sCr) and blood urea nitrogen (BUN) were increased by the CLP treatment in the rats but were blocked by the circTLK1 shRNA. The circTLK1 shRNA reduced the CLP-induced kidney injury in the rats. The circTLK1 knockdown repressed oxidation stress, inflammation, and apoptosis in the sepsis-related AKI rat model. Moreover, lipopolysaccharide (LPS) treatment increased the production of TNF-α, IL-1β, and IL-6 in the HK-2 cells, while the circTLK1 shRNA could attenuate the enhancement in the cells. Bax and cleaved caspase-3 expression was upregulated, but Bcl-2 expression was downregulated by the LPS in the HK-2 cells, in which circTLK1 depletion reversed this effect in the cells. The depletion of circTLK1 attenuated the LPS-induced apoptosis in the HK-2 cells. CircTLK1 enhanced HMGB1 expression by sponging miR-106a-5p in the HK-2 cells, and miR-106a-5p and HMGB1 were involved in circTLK1-meidated injury of LPS-treated cells. Therefore, we concluded that circTLK1 contributed to sepsis-associated AKI by regulating inflammation and oxidative stress through the miR-106a-5p/HMGB1 axis. CircTLK1 and miR-106a-5p may be employed as the potential targets for the treatment of AKI.

## Introduction

Sepsis serves as a system inflammation process and a severe disorder caused by a dysregulated reply to infection and usually results in organ failure (Faix, 2013; Cecconi et al., 2018). Sepsis has been characterized by increased sensitivity to secondary infection and low consequences resulting from a transient hyperimmune environment (Bellomo et al., 2017). Sepsis is the primary cause of mortality from infection in the world, with a broad load of about 30 million persons each year (Aslan et al., 2018). Around 50% of acute kidney injury (AKI) is associated with sepsis, as the common sepsis complication (Poston and Koyner, 2019). The chance of hospital death due to sepsis-related AKI is enormously higher than that without AKI (Fani et al., 2018). Therefore, investigation of the complicated modulation network is urgently needed for the exploration of more effective treatment strategies for AKI.

Circular RNAs (circRNAs) serve as an important kind of noncoding RNAs (ncRNAs) that play critical roles in various cellular processes, such as apoptosis and proliferation (Chaichian, 2020 #15)(Kristensen, 2019 #14). Several studies have identified that circRNAs are able to participate in the modulation of AKI (Fang et al., 2019). Meanwhile, circular RNA TLK1 (circTLK1) has presented the function of promoting cellular injury in different physiological models, including neuronal injury and myocardial ischemia–reperfusion injury (Wu et al., 2019; Song et al., 2020). But the effect of circTLK1 on AKI progression is still unreported. Furthermore, as well-identified small ncRNAs, microRNAs (miRNAs) are able to regulate their targets by specifically binding to the mRNA 3′ untranslated region (3′ UTR) (Chen et al., 2018; Pan et al., 2019; Lv et al., 2020). It has been found various miRNAs are involved in the regulation of AKI development, such as miR-21, miR-19b-3p, and miR-204 (Chen et al., 2018; Pan et al., 2019; Lv et al., 2020). MicroRNA-106a-5p (miR-106a-5p) has been found to modulate cell apoptosis and proliferation and is able to attenuate ox-LDL–related endothelial cell injury (Hu et al., 2020; Ma et al., 2020). Recently, high-mobility group box 1 (HMGB1) has been identified as the crucial positive contributor to sepsis, and HMGB1 inhibition provides a higher therapeutic window (Deng et al., 2018). Patients with sepsis undergo an inflammatory response driven by continuous or late HMGB1 release (Wei et al., 2019). Therefore, targeting HMGB1 may serve as a promising therapeutic strategy for sepsis-induced AKI (Xu et al., 2020). However, whether miR-106a-5p and HMGB1 are involved in circTLK1-mediated AKI progression remains unclear.

In this study, we explored the effect and the underlying mechanism of circTLK1 in the development of AKI. We found a new function of circTLK1 in contributing to sepsis-associated acute kidney injury by regulating inflammation and oxidative stress through the miR-106a-5p/HMGB1 axis.

## Materials and Methods

### AKI Rat Model

Wistar rats were obtained from Trophic Animal Feed High-Tech Co., Ltd, China, and cultured at 25°C under regular light/dark cycles. The sepsis-induced AKI rat model was constructed by cecal ligation and puncture (CLP) in the rats. Briefly, 1-day fasted rats were anesthetized using sodium pentobarbital (0.3%, 30 mg/kg, Solarbio, China). The 1-cm-long mouth was cut in the midline of the rats, and the cecum was found and ligated at the root to block blood supply. The ligated cecum was punctured using a suture needle through the serosal surface of the blind end of the intestine opposite to the mesentery, two holes were made, and some intestinal contents extruded. Then, the abdominal cavity was closed layer by layer using 1-0 silk thread, and the rat was immediately subcutaneously injected with 1 ml normal saline to supplement intraoperative fluid loss. The cecum was exposed without perforation in the sham groups. The rats were incubated at warm conditions for recovery and subsequent analysis. To obtain lentivirus carrying circTLK1 shRNA, 293T cells were transfected with recombinant lentiviral pLVTHM plasmids carrying circTLK1 shRNA using Lipofectamine 3000 (Thermo, United States). The supernatant was collected and used for virus suspension after 48 h of transfection. Virus titer was analyzed using the green fluorescent protein (GFP)–positive cell rate (TU/ml). After the construction of the CLP rat model, the rats were anesthetized by injecting xylazine and katemine as described in a previous report (Shi et al., 2020); the rats were injected with lentivirus carrying circTLK1 shRNA (1  ×  10^9^ TU) or control shRNA via tail veins. Animal care and method procedure were authorized by the Animal Ethics Committee of Cangzhou Central Hospital (approval number: 202003-08).

### Histological Analysis

Kidney tissues of rats were treated with 4% paraformaldehyde, decalcified in 5% nitric acid, followed by cutting into 5 μm sections. For dehydration, a set of alcohols were applied, and conventional paraffin embedding was carried out: the tissues were dehydrated using graded ethanol, embedded in paraffin, and cut into 5 μm sections. Afterward, hematoxylin and eosin (H&E) staining was performed on the sliced samples of femoral head and then observed under a microscope (BX‐42; Olympus, Tokyo, Japan). The scoring criteria from 0 to 5 were as follows: 0 = normal morphology; 1 = degeneration only without necrosis; and 2 (<25%), 3 (<50%), 4 (<75%), and 5 (>75%) = necrosis, vacuolar degeneration, tubular dilatation, and hemorrhage, respectively (Shi et al., 2020).

### Analysis of NGAL, Kim-1, sCr, and BUN in the Kidney of Rats

To collect serum, blood samples were collected from the rats and centrifuged at 5,000 g. The levels of serum creatinine (sCr) and blood urea nitrogen (BUN) were analyzed using the serum creatinine detection kit and the BUN detection kit (StressMarq Biosciences, BC, Canada), respectively. Urine samples obtained from the rats were processed by centrifuging (5 min, 600 g). The levels of Kim-1 and NGAL were measured using the ELISA kit (Cusabio Biotech, Zhengzhou, China).

### Analysis of Oxidative Stress and Inflammatory Factors

The levels of glutathione, malondialdehyde (MDA), and reactive oxygen species (ROS) in the kidney of the rats were analyzed using the Total Glutathione Assay Kit (Beyotime), Lipid Peroxidation MDA Assay Kit (Beyotime), and Reactive Oxygen Species Assay Kit (Beyotime), respectively. The activities of superoxide dismutase (SOD) were assessed using the Total Superoxide Dismutase Assay Kit (Beyotime). Kidney catalase (CAT) activities were measured using the CheKine Catalase (CAT) Activity Assay Kit (Abbkine). The relative levels of IL-1β and IL-6 were analyzed using the ELISA kit (Beyotime).

### Terminal Deoxynucleotidyl Transferase dUTP Nick-End Labeling (TUNEL)

Apoptosis in the rats was analyzed by using the TUNEL detection kit (Roche, Germany) according to the manufacturer’s guidance. Renal tissue samples were treated with paraformaldehyde (4%, 25°C) for fixation and treated with Triton X‐100 (0.2%). After that, the samples were incubated with FITC-labeled dUTP and terminal deoxynucleotidyl transferase and observed under a fluorescence microscope (Olympus, Japan). To detect the apoptosis levels of renal tissues, we analyzed the brown TUNEL-positive cells per field in each group.

### Cell Culture

Human renal tubular epithelial cell line (HK-2) and 293T cell lines were maintained in the lab and incubated at 37°C with 5% CO_2_ in RPMI-1640 (GE, United States) containing FBS (15%, GE, United States), streptomycin (0.1 mg/ml, GE, United States), and penicillin (100 units/mL, GE, United States). For the induction of the kidney injury model, the HK-2 cells were treated with lipopolysaccharide (LPS, 1 mg/L, Sigma-Aldrich, United States) for 24 h after the cell concentration reached 80%. Then, the cells were treated as indicated in the description. Lentiviral pLVTHM plasmids carrying circTLK1 shRNA, the pcDNA3.1-HMGB1 overexpression vector, miR-106a-5p mimic, and inhibitor were obtained (GenePharma, China) (GenScript, China). The transfection of the cells was performed using Lipofectamine 3000 (Thermo, United States).

### Analysis of Cell Apoptosis

About 2 × 10^5^ cells were plated on six-well dishes. Cell apoptosis was assessed by employing the Annexin V-FITC Apoptosis Detection Kit (CST, United States) following the manufacturer’s instruction. Shortly, about 2 × 10^5^ cells were collected, washed using binding buffer, and dyed at 25°C, followed flow cytometry analysis.

### Luciferase Reporter Gene Assay

Luciferase reporter gene assays were carried out by using the Dual-Luciferase Reporter Assay System (Promega, United States). The cells were transfected with pmirGLO-circTLK1 or pmirGLO-HMGB1, and miR-106a-5p mimic or control mimic by using the riboFECT™ CP Transfection Kit (RiboBio, China), followed by the analysis of luciferase activities based on the Dual-Luciferase Reporter Assay System (Promega, United States). As control, the luciferase activities of Renilla were measured.

### Quantitative Reverse Transcription-PCR (qRT-PCR)

Total RNAs were extracted using TRIZOL (Invitrogen, United States). The first-strand cDNA was manufactured according to the manufacturer’s instruction (TaKaRa, China). qRT-PCR was carried out by applying SYBR-Green (TaKaRa, China). The primer sequences were as follows: circTLK1 forward: 5′-CAG​TCA​ATG​GAG​CAG​AGA​A-3′, reverse: 5′-CCA​TTC​TTG​TTG​CCT​TTT​TG-3′; miR-106a-5p forward: 5′-GAT​GCT​CAA​AAA​GTG​CTT​ACA​GTG​CA-3′; HMGB1 forward: 5′-TGG​TAT​TTT​GGA​CTG​CGG​GG-3′, reverse: 5′-TGA​CAT​TTT​GCC​TCT​CGG​CT-3′; GAPDH forward: 5′-TGA​AGG​TCG​GAG​TCA​ACG​G-3′, reverse: 5′-TCC​TGG​AAG​ATG​GTG​ATG​GGA-3′.

### Western Blot Analysis

Total proteins were extracted from the cells using RIPA buffer (CST, United States) and quantified using the BCA Protein Quantification Kit (Abbkine, United States). The proteins at the same concentration were subjected to SDS-PAGE and transferred onto PVDF membranes (Millipore, United States), followed by incubation with 5% milk and with primary antibodies at 4°C overnight. The corresponding secondary antibodies (Boster, China) were used for incubating the membranes for 1 h at room temperature, followed by visualization using the chemiluminescence detection kit (Beyotime, China). The primary antibodies applied in this study comprised Bax (Abcam, United States), Bcl-2 (Abcam, United States), cleaved caspase-3 (Abcam, United States), TNF-α (Abcam, United States), IL-1β (Abcam, United States), IL-6 (Abcam, United States), HMGB1 (Abcam, United States), and β-actin (Abcam, United States).

### Statistical Analysis

Data were expressed as mean ± SD, and statistical analysis was conducted using GraphPad Prism 7. Unpaired Student’s *t*-test was used to compare two groups, and one-way ANOVA was used to compare among multiple groups. Fold change in qPCR was analyzed using the Mann-Whitney U test. *P* < 0.05 was considered statistically significant. The experiments were repeated at three different times.

## Results

### Depletion of circTLK1 Attenuates Sepsis-Related AKI *in Vivo*

**FIGURE 1:**
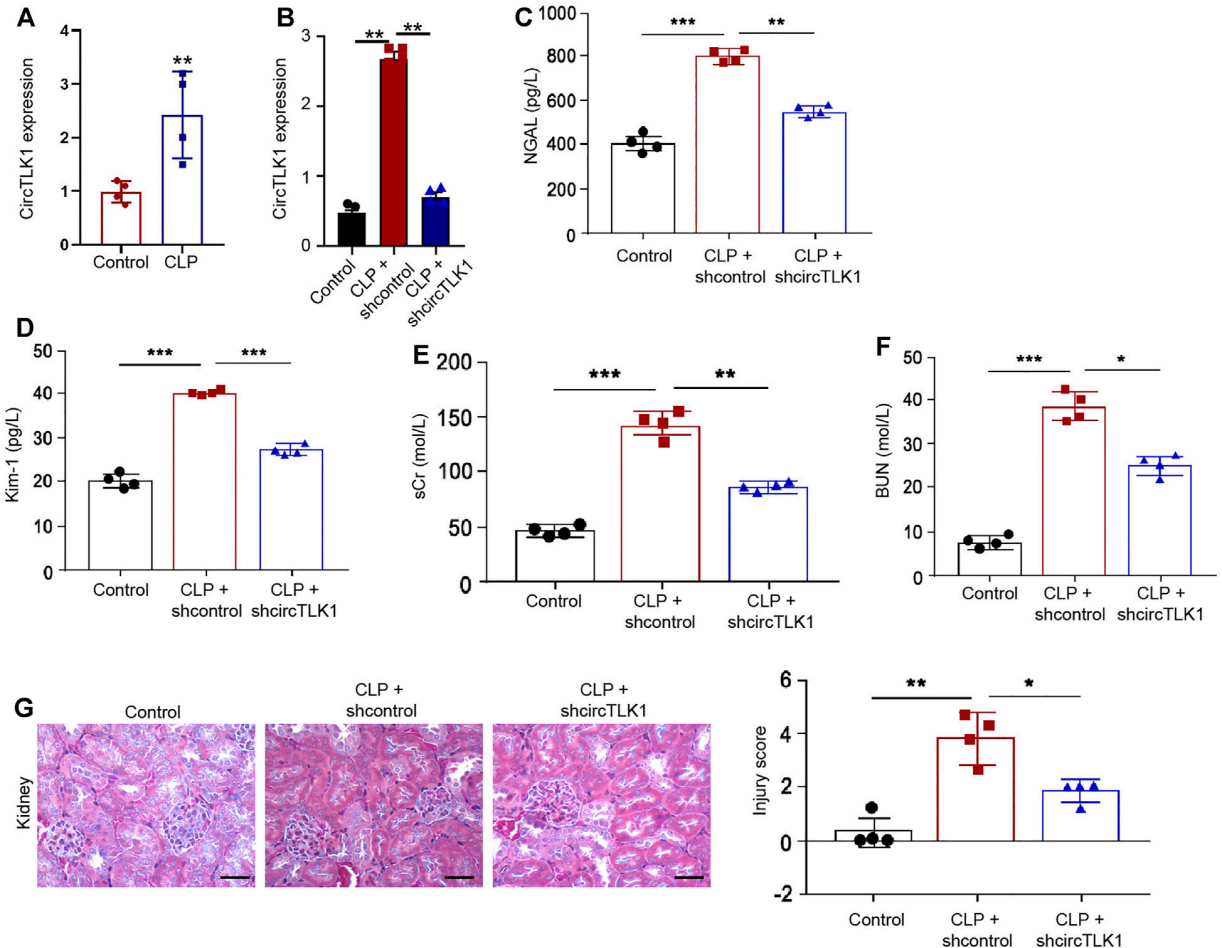
Depletion of circTLK1 attenuates sepsis-related AKI *in vivo*. **(A–G)** The CLP-induced sepsis-related AKI rat model was established in Wistar rats (*N* = 4), followed by the treatment of circTLK1 shRNA. The cecum was exposed without perforation in the sham groups. **(A**,**B)** qPCR analysis of circTLK1 expression. **(C**,**D)** Urine levels of NGAL and Kim-1. **(E**,**F)** Serum levels of sCr and BUN. **(G)** H&E staining of kidney tissues, bar = 50 μm. The experiments were replicated at least three times, mean ± SD, *P < 0.05, **P < 0.01, ***P < 0.001.

Initially, we explored the effect of circTLK1 on sepsis-related AKI in the CLP-induced rat model. To evaluate the correlation of circTLK1 with sepsis-related AKI, we detected the expression of circTLK1 in the CLP-induced rat model. circTLK1 expression was enhanced in the CLP rats relative to that in the control rats (Figures 1A,B), while the depletion of circTLK1 by shRNA repressed the expression of circTLK1 (Figure 1B). We then evaluated the effect of circTLK1 knockdown on general AKI markers, including NGAL and Kim-1 in urine and sCr and BUN in the serum of the rats. Urine levels of NGAL and Kim-1 were increased by the CLP treatment in the rats but were blocked by the circTLK1 shRNA in the system (Figures 1C,D). Meanwhile, CLP rats demonstrated enhanced serum levels of sCr and BUN, while the circTLK1 knockdown repressed this enhancement (Figures 1E,F). Then, we assessed the impact of circTLK1 depletion on kidney injury by H&E staining. circTLK1 shRNA reduced the CLP-induced kidney injury in the rats (Figure 1G).

### Depletion of circTLK1 Decreases Oxidation Stress, Inflammation, and Apoptosis in the Sepsis-Related AKI Rat Model

**FIGURE 2:**
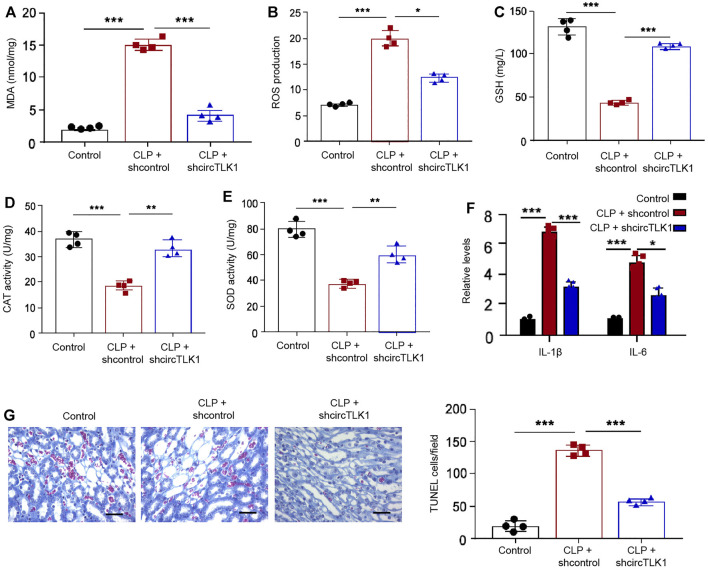
Depletion of circTLK1 decreases oxidation stress, inflammation, and apoptosis in the sepsis-related AKI rat model. **(A–G)** The CLP-induced sepsis-related AKI rat model was established in Wistar rats (*N* = 4), followed by the treatment of circTLK1 shRNA. The cecum was exposed without perforation in the sham groups. **(A)** MDA levels in the rats. **(B)** ROS production in the rats. **(C)** GSH levels in the rats. **(D)** CAT activities in the rats. **(E)** SOD activities in the rats. **(F)** IL-1β and IL-6 release in the rats. **(G)** TUNEL assays of apoptosis analysis in the rats, bar = 50 μm. The experiments were replicated at least three times, mean ± SD, *P < 0.05, **P < 0.01, ***P < 0.001.

Moreover, we further evaluated the function of circTLK1 in regulating oxidation stress and inflammation by detecting general oxidation stress markers, including MDA, ROS, GSH, and SOD, and general inflammatory cytokines, such as IL-1β and IL-6, in the CLP-induced rat model. We observed that CLP treatment enhanced the accumulation of MDA, ROS, and GSH in the rats, while circTLK1 shRNA alleviates this enhancement in the rats (Figures 2A–C). The activities of CAT and SOD were repressed by CLP treatment, but the circTLK1 depletion could increase activities in the rats (Figures 2D,E). The levels of inflammatory factors, including IL-1β and IL-6, were upregulated in the CLP rats, and the circTLK1 knockdown suppressed the upregulation in the rats (Figure 2F). Moreover, the depletion of circTLK1 attenuated CLP-induced apoptosis in the rats (Figure 2G).

### CircTLK1 Knockdown Relieves LPS-Induced Inflammation and Apoptosis in HK-2 Cells

**FIGURE 3:**
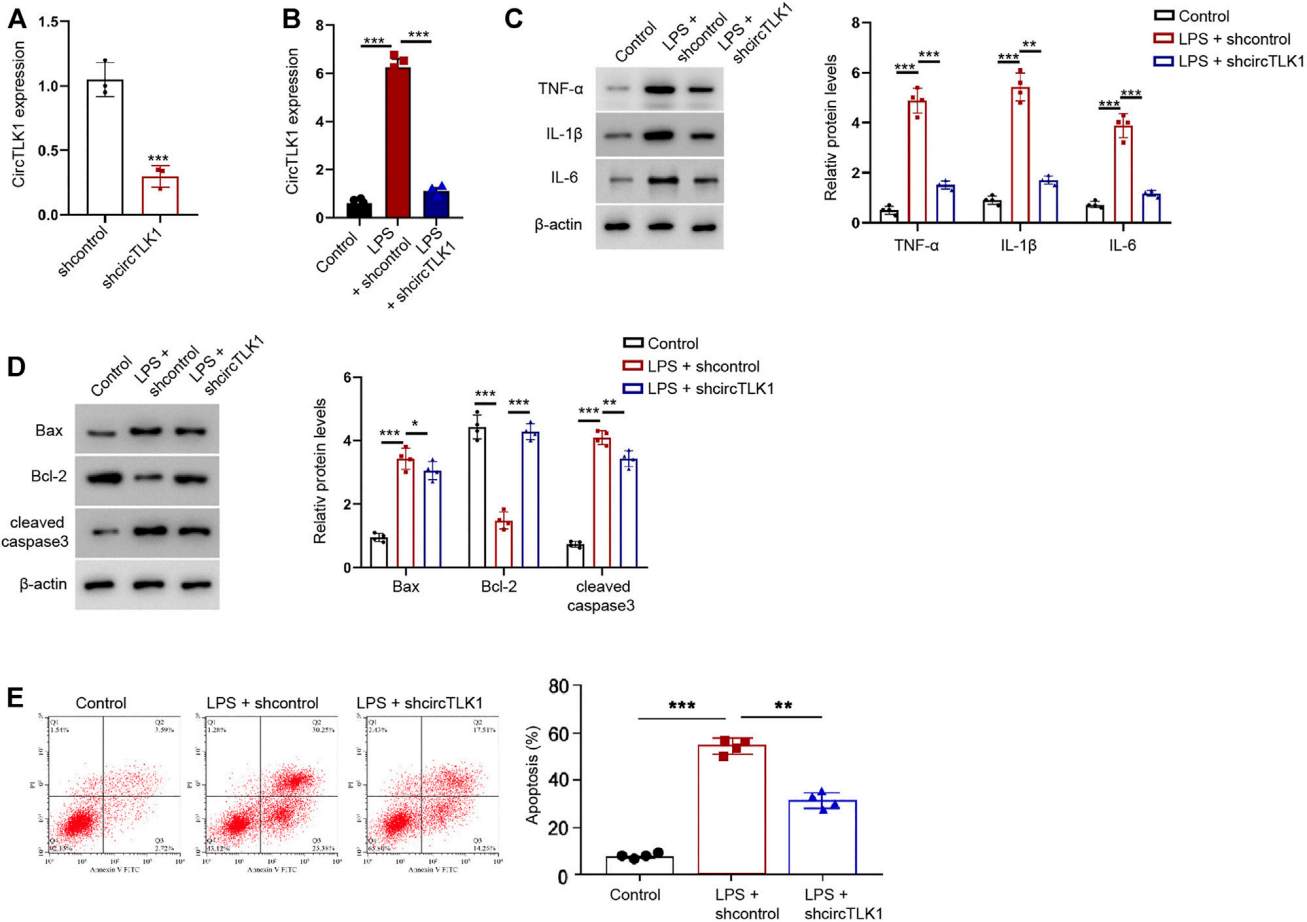
CircTLK1 knockdown relieves LPS-induced inflammation and apoptosis in the HK-2 cells. **(A)** Expression of circTLK1 was detected by qPCR in HK-2 cells treated with circTLK1 shRNA. **(B–E)** For the induction of the cell injury model, the HK-2 cells were treated with LPS (1 mg/L) for 24 h after the cell concentration reached 80%, followed by the treatment of circTLK1 shRNA. **(B)** qPCR analysis of circTLK1 expression. **(C)** Western blot analysis of TNF-α, IL-1β, and IL-6. **(D)** Western blot analysis of Bax, Bcl-2, and cleaved caspase-3. **(E)** Flow cytometry analysis of apoptosis. The experiments were replicated at least three times, mean ± SD, *P < 0.05, **P < 0.01, ***P < 0.001.

To determine the function of circTLK1 *in vitro*, we analyzed the effect of circTLK1 on AKI injury in the LPS-treated HK-2 cells, in which the LPS treatment was used to stimulate HK-2 cell injury, and in the established *in vitro* AKI model. The effectiveness of circTLK1 shRNA was validated in the HK-2 cells (Figures 3A,B). The LPS treatment enhanced the production of inflammatory factors, including TNF-α, IL-1β, and IL-6, in the HK-2 cells, while the circTLK1 shRNA attenuated the enhancement in the cells (Figure 3C). The apoptosis markers, including Bax and cleaved caspase-3 expression, were increased, but the Bcl-2 expression was decreased by the LPS in the HK-2 cells, in which circTLK1 knockdown could reverse this effect in the cells (Figure 3D). Similarly, the depletion of circTLK1 relieved LPS-induced apoptosis in the HK-2 cells (Figure 3E).

### CircTLK1 Sponges miR-106a-5p in HK-2 Cells

**FIGURE 4:**
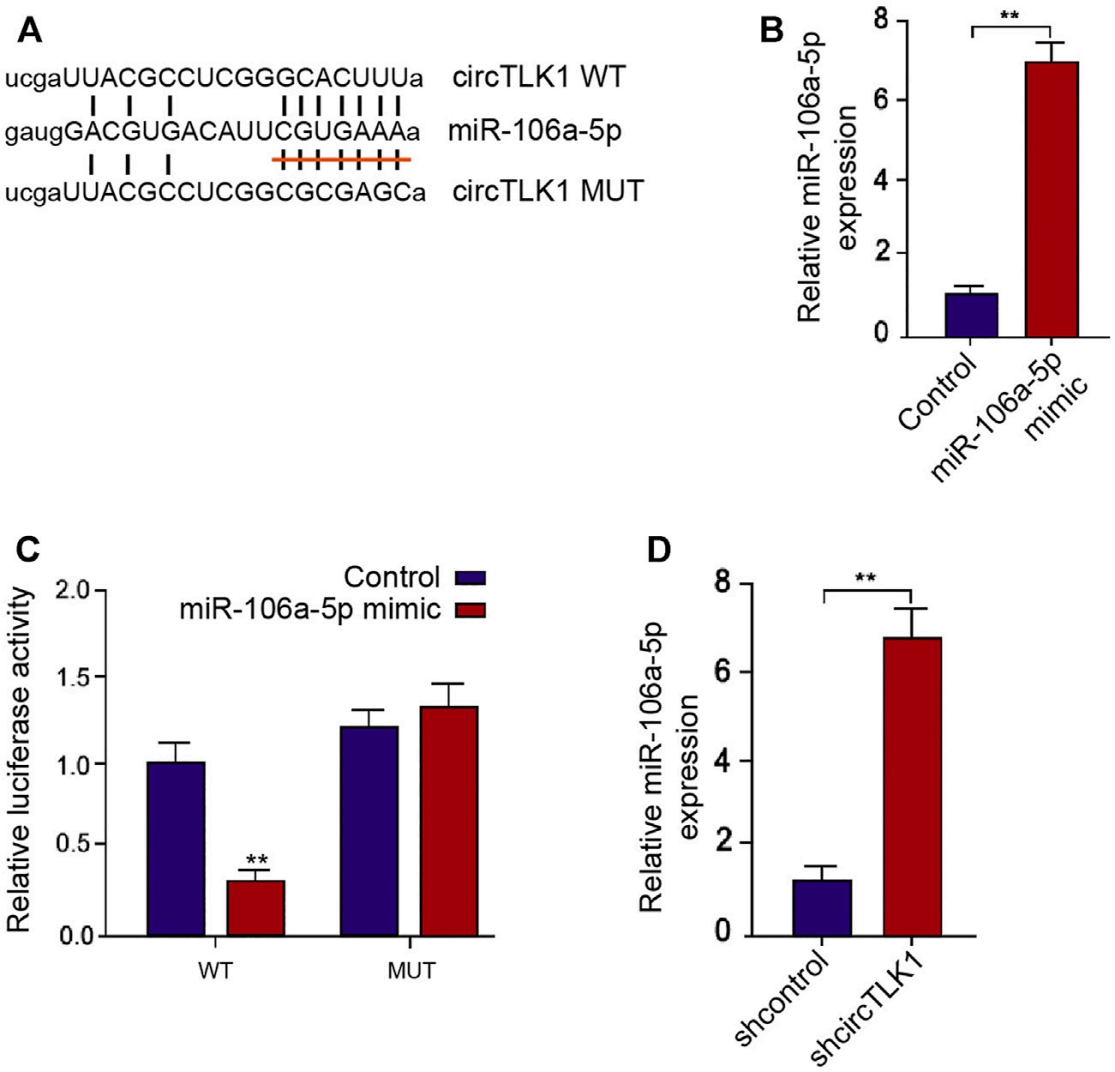
CircTLK1 sponges miR-106a-5p in HK-2 cells. **(A)** Bioinformatic analysis using ENCORI database. The sequences of miR-106a-5p, wild-type circTLK (WT), and mutant circTLK (MUT) are shown. **(B**,**C)** HK-2 cells were treated with miR-106a-5p mimic. **(B)** qPCR assays of miR-106a-5p expression. **(C)** Luciferase reporter gene assays of wild-type circTLK (WT) and mutant circTLK (MUT) luciferase activities. **(D)** HK-2 cells were treated with circTLK1 shRNA. The qPCR assays of miR-106a-5p expression, mean ± SD, **P < 0.01.

Next, we further explored the potential mechanism underlying circTLK1-meidated AKI. Mechanically, the sponge potential with miR-106a-5p of circTLK1 was identified in the bioinformatics prediction (Figure 4A). The effectiveness of miR-106a-5p mimic was validated in the HK-2 cells, and the treatment of miR-106a-5p mimic significantly reduced the circTLK1 luciferase activities in the HK-2 cells (Figures 4B,C). The circTLK1 shRNA could enhance the miR-106a-5p levels in the cells (Figure 4D).

### HMGB1 Is Targeted by miR-106a-5p in HK-2 Cells

**FIGURE 5:**
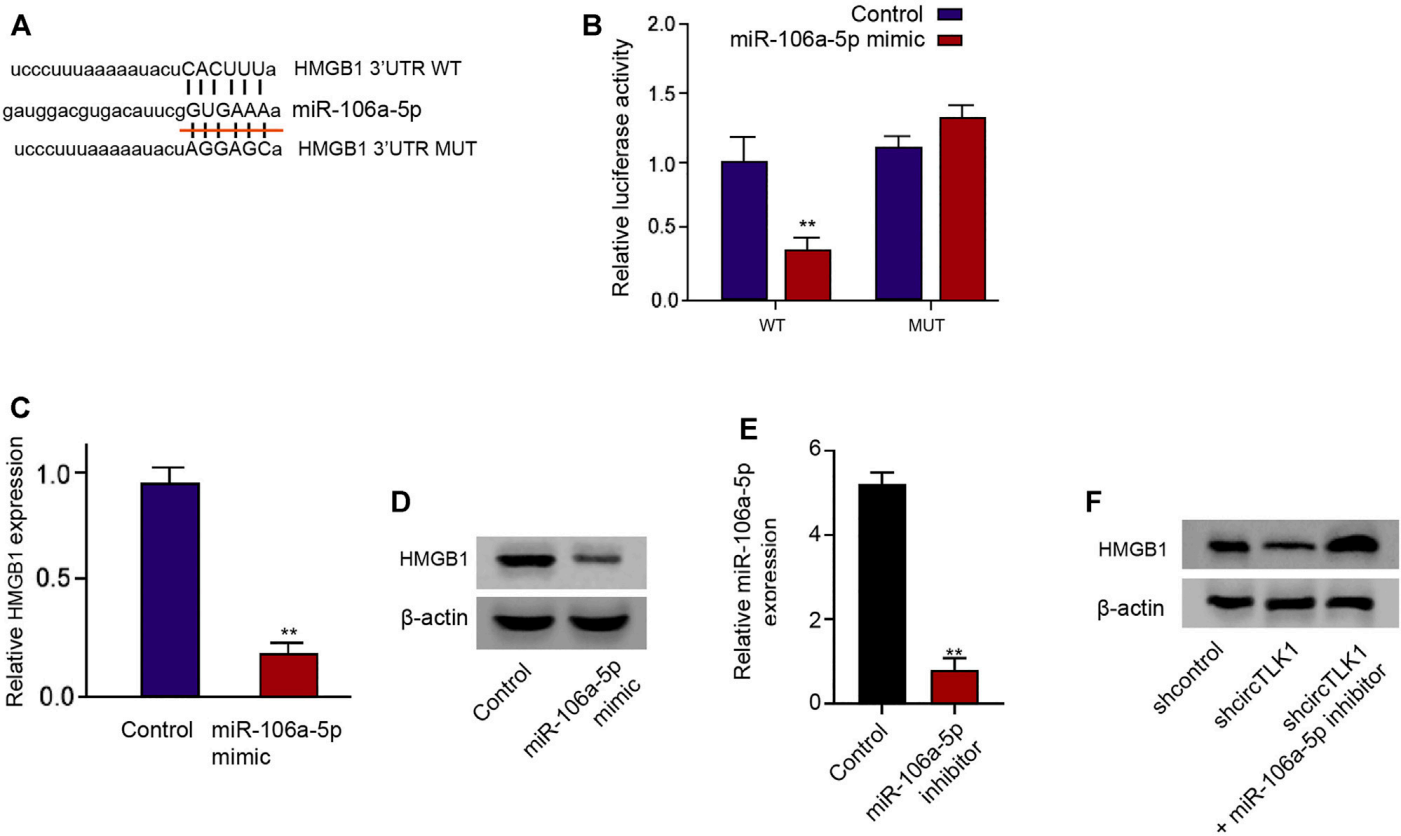
HMGB1 is targeted by miR-106a-5p in HK-2 cells. **(A)** Bioinformatic analysis using ENCORI database. The sequences of miR-106a-5p, wild-type HMGB1 3′UTR (WT), and mutant HMGB1 3′UTR (MUT) are shown. **(B–D)** HK-2 cells were treated with miR-106a-5p mimic. **(B)** luciferase reporter gene assays of wild-type HMGB1 3′UTR (WT), and mutant HMGB1 3′UTR (MUT) luciferase activities. **(C)** qPCR assays of HMGB1 expression. **(D)** Western blot analysis of HMGB1 expression. **(E)** The expression of miR-106a-5p was detected by qPCR in HK-2 cells treated with miR-106a-5p inhibitor. **(F)** HK-2 cells were treated with circTLK1 shRNA, or cotreated with circTLK1 shRNA and miR-106a-5p inhibitor. Western blot analysis of HMGB1 expression, mean ± SD, **P < 0.01.

Moreover, we tried to identify the potential target of miR-106a-5p. We also found the potential binding sites of miR-106a-5p in HMGB1 mRNA 3′UTR (Figure 5A). The luciferase activities of HMGB1 mRNA 3′UTR were decreased by miR-106a-5p mimic in the HK-2 cells (Figure 5B). Consistently, miR-106a-5p mimic reduced the mRNA and protein levels of HMGB1 in the cells (Figures 5C,D). The effectiveness of the miR-106a-5p inhibitor was verified in the HK-2 cells (Figure 5E). The depletion of circTLK1 repressed HMGB1 protein levels in the HK-2 cells, while the miR-106a-5p inhibitor could reverse this effect in the cells (Figure 5F).

### CircTLK1 Induces Sepsis-Related AKI by the miR-106a-5p/HMGB1 Axis

**FIGURE 6:**
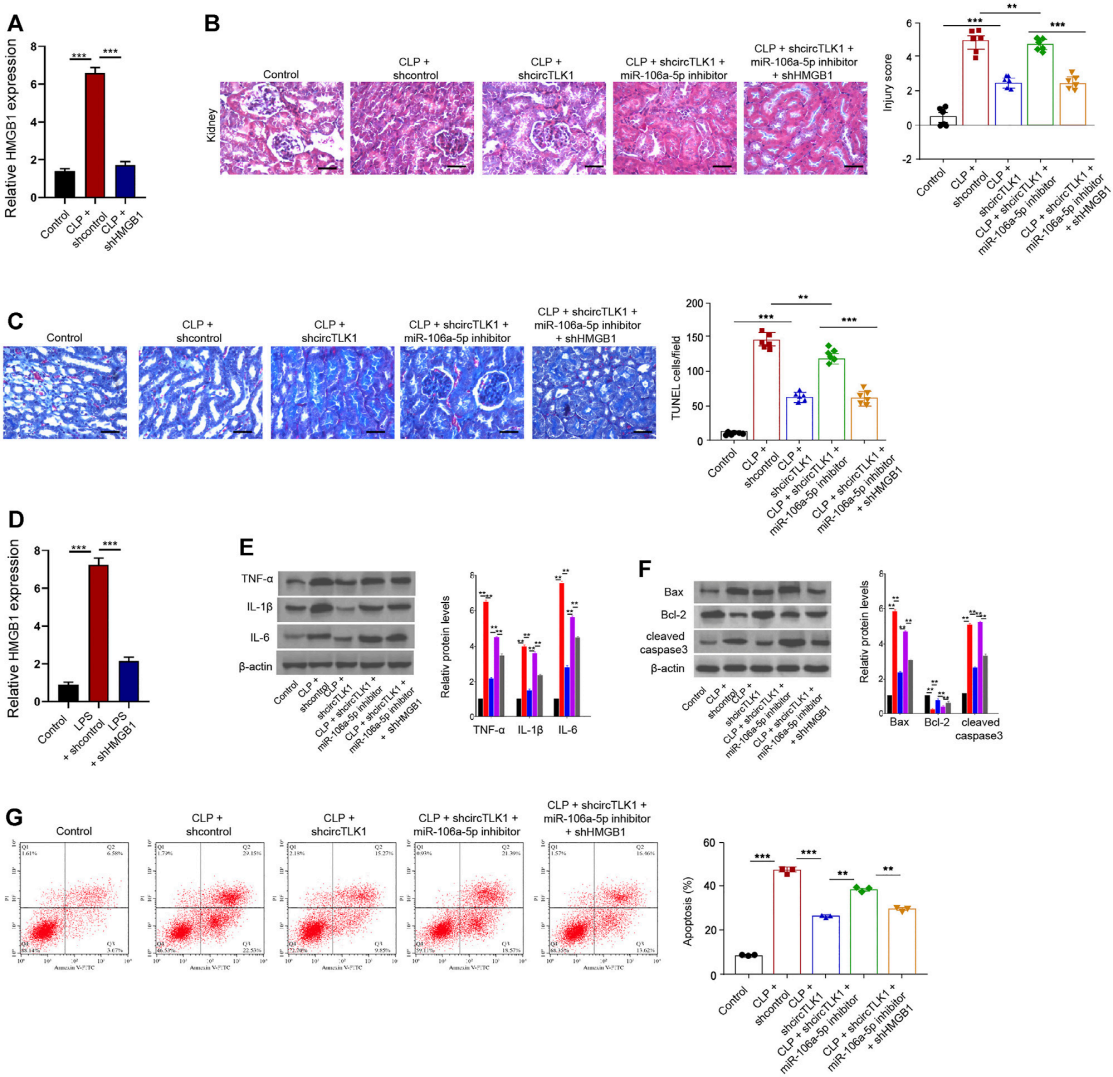
CircTLK1 induces sepsis-related AKI by the miR-106a-5p/HMGB1 axis. **(A–C)** The CLP-induced sepsis-related AKI rat model was established in Wistar rats (*N* = 6), followed by the indicated treatment. The cecum was exposed without perforation in the sham groups. **(A)** qPCR analysis of HMGB1 expression. **(B)** H&E staining of kidney tissues, bar = 50 μm. **(C)** TUNEL assays of apoptosis analysis in the rats, bar = 50 μm. **(D–G)** For the induction of the cell injury model, the HK-2 cells were treated with LPS (1 mg/L) for 24 h after the cell concentration reached 80%, followed by the indicated treatment. **(D)** qPCR analysis of HMGB1 expression. **(E)** Western blot analysis of TNF-α, IL-1β, and IL-6. **(F)** Western blot analysis of Bax, Bcl-2, and cleaved caspase-3. **(G)** Flow cytometry analysis of apoptosis, mean ± SD, **P < 0.01, ***P < 0.001.

The efficiency of HMGB1 shRNA was confirmed in the CLP rat model (Figure 6A). The CLP-induced kidney injury and apoptosis were relieved by circTLK1 shRNA, while these were enhanced by the miR-106a-5p inhibitor in the rat model, in which the depletion of HMGB1 could reverse this effect (Figures 6B,C). The effectiveness of HMGB1 knockdown by shRNA was validated in the HK-2 cells (Figure 6D). The miR-106a-5p inhibitor enhanced the circTLK1 depletion–inhibited expression of TNF-α, IL-1β, and IL-6 in the LPS-treated HK-2 cells, in which the HMGB1 depletion could reverse the phenotype (Figure 6E). Moreover, the miR-106a-5p inhibitor induced circTLK1 depletion–attenuated apoptosis of the LPS-treated HK-2 cells, while the HMGB1 knockdown reversed this effect in the cells (Figures 6F,G).

## Discussion

Sepsis-associated AKI is a severe disease with enhanced inflammation and oxidation stress. CircRNAs play critical roles in multiple pathological conditions. In the present study, we uncovered that circTLK1 contributed to sepsis-associated AKI by regulating inflammation and oxidative stress through the miR-106a-5p/HMGB1 axis.

The potential functions of circRNAs in AKI have been identified. CircVMA21 relieves oxidative stress and inflammation of sepsis-related AKI *via* targeting miR-9-3p/SMG (Shi et al., 2020). CircPRKCI attenuates LPS-induced injury of HK-2 cells by modulating miR-545/ZEB2 signaling (Shi et al., 2020). Circ_0068,888 relieves LPS-induced injury of HK-2 cells by targeting miR-21-5p (Wei et al., 2021). Circ_0114427 is able to regulate inflammation in the AkI model (Cao et al., 2020). Circ_0068,888 reduces LPS-induced injury of HK-2 cells by sponging miR-21-5p (Wei et al., 2021). Most of these studies only focused on the cell model. These studies indicate the critical roles of circular RNAs in regulating AKI. Meanwhile, some investigations have reported that other noncoding RNAs are involved in the modulation of AKI. For example, it has been reported that long noncoding RNA DANCR inhibits LPS-stimulated septic AKI by targeting miR-214 (Zhao et al., 2020). Long noncoding RNA HOXA-AS2 regulates miR-106b-5p to reduce sepsis-related AKI (Wu et al., 2020). Our data showed that the circTLK1 expression was elevated in the CLP rats. The depletion of circTLK1 attenuated sepsis-related AKI in the CLP rat model. The circTLK1 knockdown attenuated the CLP-induced kidney injury score in the rats. The depletion of circTLK1 relieved oxidation stress, inflammation, and apoptosis in the sepsis-related AKI rat model. Meanwhile, circTLK1 knockdown reduced LPS-induced inflammation and apoptosis in HK-2 cells. These data suggest that circTLK1 contributes to the oxidation stress, inflammation, and apoptosis during sepsis-associated AKI *in vivo* and *in vitro*, presenting a novel function of circTLK1 in AKI. Moreover, targeting circTLK1 may be applied as a therapeutic strategy for sepsis-associated AKI. The clinical significance of circTLK1 in sepsis-associated AKI should be explored in future investigations.

Moreover, it has been found that miR-128-3p enhances inflammation by repressing NRP1 levels in sepsis-induced AKI (Wang et al., 2020). Long noncoding RNA NEAT1 modulates sepsis-related AKI by regulating miR-204/NF-κB signaling (Chen et al., 2018). MiR-106a promotes the sepsis-associated AKI *via* downregulating THBS2 *in vivo* (Shen et al., 2019). Furthermore, miR-129-5p attenuates LPS-stimulated AKI by regulating HMGB1/TLRs/NF-κB signaling (Huang et al., 2020). TNF-α/HMGB1 signaling is involved in proptosis in AKI (Wang et al., 2020). SIRT1-regulated deacetylation of HMGB1 inhibits sepsis-induced AKI (Wei et al., 2019). We found that circTLK1 upregulated HMGB1 expression by sponging miR-106a-5p in the HK-2 cells, and miR-106a-5p and HMGB1 were involved in circTLK1-mediated sepsis-induced AKI in the cells. These data elucidate the correlation of circTLK1 with miR-106a-5p and HMGB1 in the sepsis-induced AKI, presenting a new mechanism of sepsis-induced AKI development.

In conclusion, we identified that circTLK1 contributed to sepsis-associated AKI by regulating inflammation and oxidative stress through the miR-106a-5p/HMGB1 axis. CircTLK1 and miR-106a-5p may be employed as the potential targets for the treatment of AKI.

## Data Availability

The original contributions presented in the study are included in the article/Supplementary Material. Further inquiries can be directed to the corresponding author.
